# Comparison of Fungal and Non-Fungal Rhinosinusitis by Culture-Based Analysis

**DOI:** 10.3390/jpm13091368

**Published:** 2023-09-11

**Authors:** Chih-Hung Cha, Wei-Chih Chen, Yu-Ming Wang, Shao-Chun Wu, Tai-Jan Chiu, Ching-Nung Wu, Yinshen Wee, Ching-Shuen Wang, Yao-Hsu Yang, Sheng-Dean Luo

**Affiliations:** 1 Department of Otolaryngology, Kaohsiung Chang Gung Memorial Hospital, Chang Gung University College of Medicine, Kaohsiung 833, Taiwan; d070025@cgmh.org.tw (C.-H.C.);; 2 Department of Radiation Oncology and Proton & Radiation Therapy Center, Kaohsiung Chang Gung Memorial Hospital, Chang Gung University College of Medicine, Kaohsiung 833, Taiwan; 3 Department of Anesthesiology, Kaohsiung Chang Gung Memorial Hospital, Chang Gung University College of Medicine, Kaohsiung 833, Taiwan; 4 Graduate Institute of Clinical Medical Sciences, College of Medicine, Chang Gung University, Taoyuan 333, Taiwan; 5 Department of Hematology-Oncology, Kaohsiung Chang Gung Memorial Hospital, Chang Gung University College of Medicine, Kaohsiung 833, Taiwan; 6 Department of Pathology, University of Utah, Salt Lake City, UT 84112, USA; 7 School of Dentistry, College of Oral Medicine, Taipei Medical University, Taipei 110, Taiwan; chingshuenwang@tmu.edu.tw; 8 Department of Traditional Chinese Medicine, Chang Gung Memorial Hospital, Chiayi 613, Taiwan; 9 Health Information and Epidemiology Laboratory of Chang Gung Memorial Hospital, Chiayi 613, Taiwan; 10 School of Traditional Chinese Medicine, College of Medicine, Chang Gung University, Taoyuan 333, Taiwan

**Keywords:** fungal sinusitis, chronic rhinosinusitis, sinus surgery, rhinosinusitis

## Abstract

Background: Incidence of fungal rhinosinusitis has increased in recent few years. We investigated the differences in microbiological findings between patients with fungal and non-fungal rhinosinusitis by growing microbiological cultures from samples obtained from sinus surgery. Methods: Using the Chang Gung Research Database, we enrolled all chronic rhinosinusitis (CRS) patients who had ever undergone sinus surgery from 2001 to 2019 and had microbiological culture during sinus surgery. Enrolled patients were divided into fungal and non-fungal groups, based on fungal culture and surgical pathology. Results: A total of 898 patients were diagnosed with fungal rhinosinusitis and 2884 with non-fungal rhinosinusitis. The fungal group had a higher age distribution (56.9 ± 13.1 vs. 47.0 ± 14.9), a larger proportion of females (62.4% vs. 37.0%), more unilateral lesions (80.4% vs. 41.6%), a lower incidence of the need for revision surgery (3.6% vs. 6.0%, *p* = 0.004), and a higher proportion of *Pseudomonas aeruginosa* in the culture (14.3% vs. 4.6%, *p* < 0.001). Conclusions: This large-scale study showed that *Pseudomonas aeruginosa* are more commonly found in patients with fungal rhinosinusitis and in patients who needed revision surgery, suggesting that efforts aimed at eliminating *Pseudomonas* are needed in order to improve the disease outcomes of patients with fungal rhinosinusitis.

## 1. Introduction

Chronic rhinosinusitis (CRS) is a multifactorial disease complicated by interactions between environmental factors, pathogens, and the host immune system [1]. The sinuses harbor a complex bacterial and fungal microbiome, but their roles in sinus microenvironment are still poorly understood. Microbiome has long been considered to play a role in pathogenesis, or in exacerbations and progression of the inflammatory process. Recent microbiome studies have revealed a loss of bacterial diversity and enrichment of sinus pathobionts in the microbial profiles of CRS patients [2,3,4].

Fungal rhinosinusitis is a distinct type of rhinosinusitis with increasing incidence in recent few years [5]. Noninvasive fungal rhinosinusitis includes allergic fungal rhinosinusitis (AFRS) and fungus ball (FB) [6]. AFRS is a kind of CRS with nasal polyps (CRSwNP) characterized by eosinophilic mucin and type I hypersensitivity, accounting for less than 5% of CRS cases [7]. AFRS is considered to be extremely rare in Taiwan, with only few cases reported throughout the literature [8]. Fungal balls (FB) are non-invasive collections of fungal mycelia in sinuses. Instead of the fungus itself, the host immune status usually determines the presentation of the disease [9]. Host immune response and interactions between bacteria and fungi contribute to different manifestations of rhinosinusitis [10,11,12]. Mycological culture seldom yields a fungus, only in less than 40% of cases. Complex microbial interactions with bacterial species may weaken and inhibit the growth of fungus [5].

Surgery is the mainstream treatment for fungal rhinosinusitis with high successful rate [13]. However, some patients suffer from recurrent rhinosinusitis after the sinus surgery. The recurrence rate of the fungal ball has been reported to be about 4% [14]. Dysfunction of the sinus cavity and long-term postoperative mucostasis occurred in 18% of the patients [15]. The concomitant use of antibiotics is suggested for the perioperative care of fungal rhinosinusitis, but there are few published data or antibiotics susceptibility reports to guide the clinical use. Recognition of the microbiology of CRS is of great importance when selecting an antimicrobial treatment. This study seeks to compare the microbiology of fungal rhinosinusitis with non-fungal CRS. 

Previous studies emphasized the comparison of bacteriologic profiles of CRSwNP and CRS without nasal polyps (CRSsNP). However, the microbiology of fungal rhinosinusitis has rarely been discussed. In this study, we used the Chang Gung Research Database (CGRD), a multi-medical institutional database in Taiwan, to investigate the differences in microbiological findings between patients with fungal and non-fungal rhinosinusitis. The CGRD is derived from the original medical records of Chang Gung Memorial Hospital (CGMH), which comprises seven medical institutes in Taiwan. CGMH, with its 10,070 beds, admits more than 280,000 patients annually. Moreover, the CGRD offers extensive overall and disease-specific coverage of Taiwan [16,17]. Studies conducted using the CGRD have been recognized for their high quality and positive impact on healthcare in Taiwan. To our knowledge, this is the largest cohort to compare fungal rhinosinusitis with non-fungal rhinosinusitis in terms of the differences in microbiological findings.

## 2. Materials and Methods

### 2.1. Data Source: The CGRD

We derived sinus culture results from the CGRD to assess to microbiological features of fungal rhinosinusitis. Due to the de-identified nature of the CGRD and observational design, this study was exempt from informed consent. The study was conducted in accordance with the Declaration of Helsinki and approved by the Institutional Review Board (reference numbers: 202001137B0 and 202001137B0C601) of CGMH. 

### 2.2. Study Groups—Data Extraction

Using the CGRD from 2001 to 2019, we enrolled adult patients older than 18 years old with CRS who had ever undergone sinus surgery and had microbiological culture during the sinus surgery. CRS and nasal polyps-related diagnostic International Classification of Disease codes included ICD-9 (471, 471.0–471.1, 471.8–471.9, 473, 473.0–473.3, 473.8–473.9) and ICD-10 (J32, J32.0–J32.4, J32.8–J32.9, J33, J33.0–J33.1, J33.9). The sinus surgery code was based on the reimbursement system of the Bureau of National Health Insurance of Taiwan, with both external and endonasal approaches in the ICD-10 procedure coding system. We further documented the pathological diagnosis of nasal polyps and fungal infection based on the codes of Systematized Nomenclature of Medicine-Clinical Terms (SNOMED CT). 

Patients with a positive fungal culture or diagnosis of fungal infection by the surgical pathology were classified as the fungal group [13]. Otherwise, the rest were classified as the non-fungal group. Bacterial cultures were generally collected from swab samples obtained from both middle meati using a cotton-tipped stick, as the swab sampling method, widely used among rhinologists in Taiwan, is the easiest way to collect the mucopurulent discharge from the ostiomeatal complex, which is the treatment target of CRS [18]. All the culture swabs were routinely incubated and examined in the laboratories of CGMH for at least seven days. The culture rate was calculated as the number of specimens with positive culture divided by the number of all specimens. As AFRS is rare in Taiwan, no eosinophilic mucin was found among all the pathology report. 

We extracted data of the laboratory tests and microbiological cultures from the CGRD database. The need for revision sinus surgery was also documented. A revision surgery was indicated for persistence of symptoms with objective CT and endoscopic evidence of recurrent disease, after optimal medical therapy [13]. 

The following comorbidities were defined according to ICD-9 and ICD-10 codes: asthma, chronic obstructive pulmonary disease (COPD), chronic kidney disease (CKD), end-stage renal disease (ESRD), cerebrovascular accident (CVA), coronary artery disease (CAD), liver cirrhosis, diabetes mellitus, and hypertension. These ICD codes were taken into account if they were documented more than twice on the medical record of outpatient visits, or at least once recorded in the admission note. Allergy was defined as at least one positive result of total IgE, Phadiatop, Mast, or CAP test. 

The exclusion criteria were as follows: patients diagnosed with invasive fungal rhinosinusitis, sinonasal tumors, and history of head and neck malignancy. Invasive fungal rhinosinusitis was defined by evidence of fungal invasion on a CT scan, such as orbital complications or skull base destruction (*N* = 27), and by the use of anti-fungus agents after the sinus surgery (*N* = 66) on the medical records in the database, according to consensus on diagnosis and treatment of invasive fungal rhinosinusitis [19,20]. We excluded patients with organ transplantation and autoimmune disorders (e.g., systemic lupus erythematosus, rheumatoid arthritis, juvenile idiopathic arthritis, Sjögren’s syndrome, polymyositis, and pemphigus) because the use of immune modulators might lead to immunocompromised status and increase risk of invasive fungal rhinosinusitis. 

### 2.3. Statistics Analysis

The demographic data and comorbidities of patients were compared between the fungal and the non-fungal group by Pearson’s chi-squared test and independent t-test. To validate the differences in microbiological findings, laboratory results, and the need for revision surgery between the two groups, multivariable analysis was applied to evaluate the Odds ratios (ORs) of fungal rhinosinusitis. In the fungal group, subgroup analysis was performed to compare those who needed a revision surgery to those who did not. A *p*-value < 0.05 was considered statistically significance. For continuous variables, the data were expressed as mean ± standard deviation. All statistical analyses were performed using SAS 9.4 and SPSS software (PASW Statistics for Windows, version 25.0; IBM Corp., Armonk, NY, USA).

## 3. Results

### 3.1. Demographics Data

**Figure 1:**
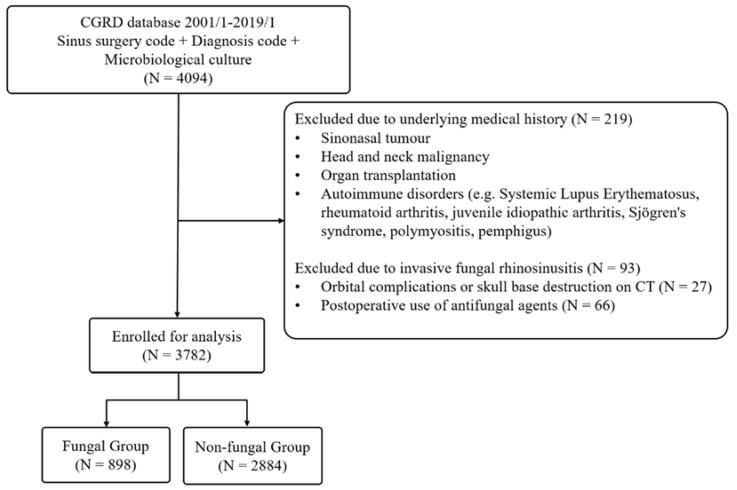
Demographic characteristics of fungal group and non-fungal group.

A total of 4094 patients were collected from the database. Altogether, 219 patients were excluded because of past medical histories or autoimmune disorders, and 93 patients were excluded because of invasive fungal rhinosinusitis (Figure 1). A total of 3782 patients were enrolled in this study. Fungal and non-fungal rhinosinusitis were diagnosed in 898 (23.7%) and 2884 (76.3%) patients, respectively. In the clinical characteristics listed in Table 1, the fungal group had a higher age distribution (56.9 ± 13.1 vs. 47.0 ± 14.9, *p* < 0.001), a larger proportion of females (62.4% vs. 37.0%, *p* < 0.001), more unilateral lesions (80.4% vs. 41.6%, *p* < 0.001), and fewer nasal polyps (18.5% vs. 47.6%, *p* < 0.001). Moreover, the fungal group had significantly more comorbidities, with a higher incidence of CKD (4.7% vs. 2.2%, *p* < 0.001), CAD (9.2% vs. 4.3%, *p* < 0.001), diabetes mellitus (13.6% vs. 7.5% *p* < 0.001), and hypertension (24.3% vs. 13.4%, *p* < 0.001) than the non-fungal group. 

### 3.2. Microbial Culture Spectrum

**Figure 2:**
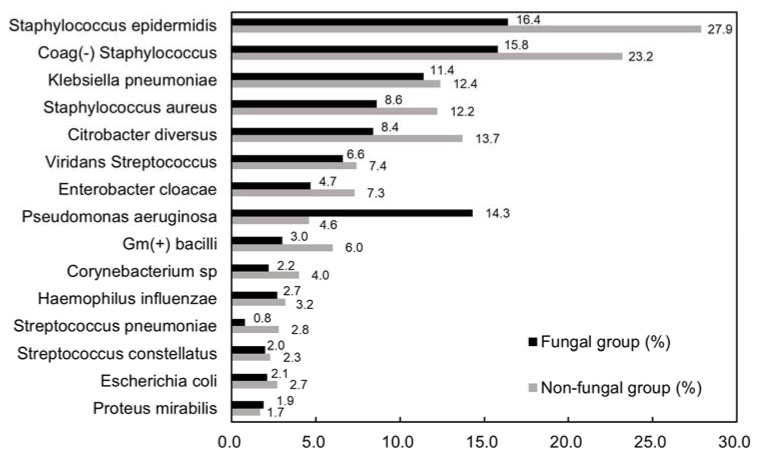
Bacteriology of aerobes in two groups.

Figure 2 shows the most common growth of aerobes in both groups. The top three species in the fungal group were *Staphylococcus epidermidis* (16.4%), coagulase-negative *Staphylococcus* (15.8%), and *Pseudomonas aeruginosa* (*P. aureus*, 14.3%). The top three species in the non-fungal group were *Staphylococcus epidermidis* (27.9%), coagulase-negative *Staphylococcus* (23.2%), and *Citrobacter diversus* (13.7%). 

**Figure 3:**
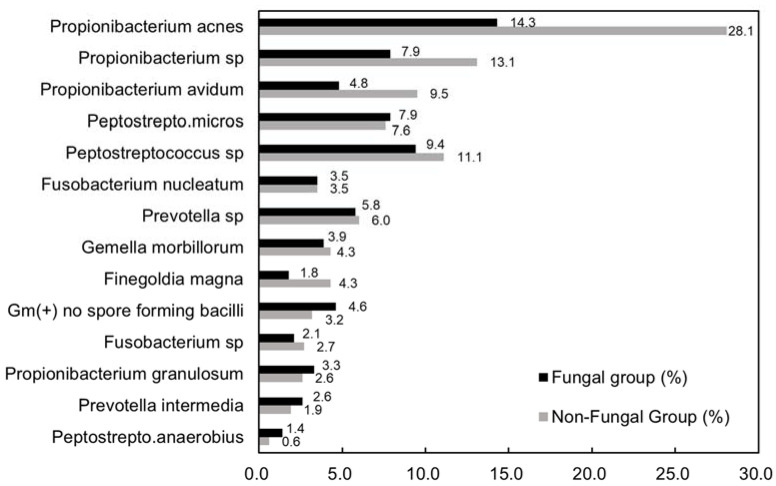
Bacteriology of anaerobes in two groups.

Figure 3 shows the most common growth of anaerobes in both groups. The top three species in the fungal group were *Propionibacterium acnes* (14.3%), *Peptostreptococcus* sp. (9.4%), and *Propionibacterium* sp. (7.9%). The top three species in the non-fungal group were *Propionibacterium acnes* (28.1%), *Propionibacterium* sp. (13.1%), and *Peptostreptococcus* sp. (11.1%). There was no significant difference in anaerobes species between the two groups. 

**Figure 4:**
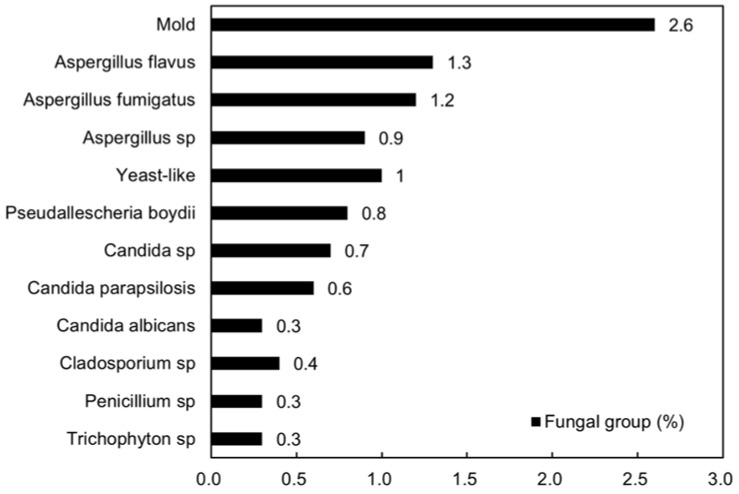
Results of fungal culture.

Figure 4 shows the most prevalent fungi identified by culture, with mold (2.6%), yeast-like species (1%), and *Aspergillus* sp. (3.4%) on the top of the list. Among those with positive fungal cultures, coagulase-negative *Staphylococcus* and *Staphylococcus epidermidis* were the most frequent concurrent bacterial cultures, followed by *Propionibacterium* sp., *Pseudomonas aeruginosa*, and *Corynebacterium* sp. 

### 3.3. Disease Characteristics

Table 2 presents the laboratory data and ratio of patients receiving revision surgery. The positive culture rate for *P. aureus* and *Staphylococcus aureus* (*S. aureus*) was further compared because of the pathogenic nature of the two species. The fungal group had a lower white blood cell count (6.7 ± 1.9 vs. 7.5 ± 2.4, *p* < 0.001) and a lower percentage of eosinophils (2.2 ± 1.9% vs. 2.7 ± 2.4%, *p* < 0.001). Fewer patients received revision surgery in the fungal group than in the non-fungal group (N = 32, 3.6% vs. 6.0%, *p* = 0.004). 

The non-fungal group tended to yield more aerobes in the culture results, with a higher positive culture rate for *S. aureus* (12.2% vs. 8.6%, *p* < 0.05). There was no statistical difference in the positive culture rate of methicillin-resistant *Staphylococcus aureus* (MRSA). Nevertheless *P. aeruginosa* had a greater prevalence in the fungal group than in the non-fungal group (14.3% vs. 4.6%, *p* < 0.001). 

### 3.4. Odds Ratios of Fungal Rhinosinusitis

Table 3 shows the odds ratios (ORs) of multiple variables in the fungal group. The culture of *P. aeruginosa* showed greater odds for fungal rhinosinusitis (adjusted OR = 2.441, 95% confidence interval, CI: 1.781–3.347, *p* < 0.001). By contrast, the fungal group showed less of a likelihood to have a *S. aureus*–positive culture (OR = 0.726, 95% CI: 0.530–0.994, *p* = 0.046). 

### 3.5. Revision Surgery in the Fungus Group

In the subgroup analysis of the fungal group, there were no significant differences between the two subgroups in terms of age, sex, nasal polyps, and laboratory data (Table 4). Patients in the revision surgery subgroup were younger (48.7 ± 13.3 vs. 57.2 ± 13.0, *p* < 0.001), had a lower incidence of CKD (3.1% vs. 1.3%, *p* < 0.001), CAD (6.3% vs. 9.4%, *p* < 0.001), diabetes mellitus (6.3% vs. 13.9%, *p* < 0.001), and hypertension (9.4% vs. 24.8%, *p* < 0.001), a greater ratio of *P. aeruginosa* (28.1% vs. 13.7%, *p* < 0.001), and a lower ratio of *S. aureus* (6.3% vs. 8.7%, *p* = 0.003) in the aerobic culture. In Table 5, the culture of *P. aeruginosa* showed greater odds for a revision surgery (adjusted OR = 4.056, 95% CI: 1.572–10.460, *p* < 0.01). 

## 4. Discussion

Microbiology is considered either a cause of CRS or a driver of exacerbation. This study used the Chang Gung multi-institute database to retrospectively investigate and compare sinonasal cultures during sinus surgery between patients with fungal and non-fungal rhinosinusitis. 

The patients in the fungal group were older, predominantly female, had a larger proportion of unilateral lesions, and fewer nasal polyps, which is consistent with the results of previous epidemiological studies [21]. In line with our results, previous microbiome studies have shown that the major isolated facultative aerobic and anaerobic bacteria were coagulase-negative *Staphylococcus* and *Propionibacterium* sp., which are commensal bacteria in the upper aerodigestive tract. *S. epidermidis* and coagulase-negative *Staphylococcus* were commonly cultured from the sinus discharge, but some regarded it as contaminant rather than a true pathogen due to the frequent discovery in normal subjects and the possible contamination from the nasal vestibule caused by our swab sampling method. Nasal bacterial culture rates were estimated to range from 30% to 60%, and our research showed similar results. 

Bacterial co-infection has been reported in 68.0% to 73.4% of patients with fungal ball rhinosinusitis [22]. Culture-based studies have revealed that co-infection of *Staphylococcus* spp., *Streptococcus* spp., *P. aeruginosa*, and *Haemophilus* sp. is common in patients with fungal rhinosinusitis [11,23,24]. *P. aeruginosa* was discovered in 8.2% of fungal ball patients who underwent Caldwell–Luc procedure [25] and in one of 32 patients with aspergillus maxillary sinus fungus ball [26]. Lu et al. [27] conducted the first study on bacterial communities in fungal rhinosinusitis using the 16s rRNA technique. *Haemophilus* sp. and *Pseudomonas* spp. are highly prevalent and abundant in patients with fungal rhinosinusitis. Another 16S rRNA study showed that *Pseudomonas spp*. has a greater prevalence in allergic fungal rhinosinusitis (AFRS) (28.6%) than in CRSsNP (15.2%) and CRSwNP (23.6%) [28]. Our culture-based study demonstrated the unique microbiological features of fungal rhinosinusitis in a much larger patient population. 

The non-fungal group tend to yield more aerobes than the fungal group, except for *P. aeruginosa*, which had a greater prevalence in the fungal group. In the subgroup analysis, we found that *P. aeruginosa* was also more prevalent in the revision surgery subgroup (28.1% vs. 13.7%, *p* < 0.001), and this subgroup was associated with more comorbidities such as CAD, diabetes, and hypertension. The culture of *P. aeruginosa* showed greater odds for fungal rhinosinusitis (OR = 2.441) and for revision surgery in the fungal group (OR = 4.056). *P. aeruginosa* was one of the most predominant microbiology species that may contribute to acute exacerbations of CRS [29]. Due to its capacity to resist antibiotics and to form biofilm, *P. aeruginosa* is thought to play a role in recalcitrant CRS. From an in vivo model of fungal sinusitis in sheep, fungal biofilm could form only after co-inoculation with *S. aureus* or *P. aeruginosa* [30]. Bacterial toxins from *S. aureus* and *P. aeruginosa* are capable of inhibiting the ciliary movement of epithelial cells, which promotes both bacterial and fungal biofilm formation. By including multiple bacterial and/or fungal species in a single community, biofilms provide theses microorganisms with resistance to antimicrobial agents. 

Both *S. aureus* and *P. aeruginosa* are biofilm-forming pathogens, but only *P. aeruginosa* seems to contribute to neo-osteogenesis in CRS [31]. Sinonasal mucosa that bacterial biofilm adheres to would release inflammatory cytokines and lead to osteitis and neo-osteogenesis [32]. Osteitis was more frequently observed in 72% to 95% of patients with fungal ball rhinosinusitis than in other types of CRS [22,33]. We reviewed 32 cases in the fungal group that required revision surgery; the diseased sinuses were different from the previous surgery, and half of them did not show evidence of fungal infection during the revision surgery. The recurrent disease was not related to surgical failure and fungal ball infection but associated with concurrent bacterial infection. Based on this fact, we postulate that biofilm of *P. aeruginosa* and fungus, for example, Aspergillus fumigatus, triggered diffuse inflammation of the sinonasal environment and caused recurrent disease after sinus surgery. Further researches are needed to investigate the role of osteitis in the pathogenesis of fungal rhinosinusitis and the *P. aeruginosa* biofilm.

Antibiotics are generally used in acute exacerbations of CRS to relieve symptoms and to prevent orbital complications [29]. CRS with positive nasal bacterial culture seems to have worse surgical outcomes and a higher recurrence rate than those with negative results [34]. Positive bacterial culture from paranasal sinus discharge has positive correlation with the severity of nasal symptoms. Therefore, efforts aimed at identifying nasal bacteria using microbiological culture are needed in order to improve the disease outcomes. In some pilot studies, *P. aeruginosa* bacteriophages successfully removed ex vivo biofilms formed by *P. aeruginosa* isolates from CRS patients [35,36]. Anti-Pseudomonas agents and bacteriophages may be used as adjuvant treatments for patients with a positive culture of *P. aeruginosa* to achieve long-term control of fungal rhinosinusitis. Based on our results of the bacterial culture, we suggest eliminating *P. aeruginosa* to prevent rhinosinusitis-related complications and to reduce the risk of disease recurrence after sinus surgery in patients with fungal rhinosinusitis. 

This study has several limitations. First, as this was a retrospective, observational study, the methods and anatomical sites used to collect the culture could not be well controlled. Even though most of the rhinologists of our authors performed the culture during sinus surgery from the pus over the ostiomeatal complex by swab samples, some surgeons may have collected the cultures by other methods that could not be well clarified due to the retrospective design. Sinus surgery without microbiological culture could not be enrolled during the data extraction. Second, the study focused on patients who failed optimal medical therapy and required sinus surgery. We lacked the results of the sinonasal cultures from the normal population and non-surgical CRS patients. Third, treatment before sinus surgery may act as a confounding factor, as uses of antibiotics may alter the sinus microbiome. Finally, we could only analyze the most dominant bacteria from a culture and could not assess the diversity and abundance of other species.

Despite the limitations, this study was performed from a cohort of 3782 patients, which reduces the sampling variability for microbiological cultures. In addition, compared to studies of the 16S rRNA technique, intraoperative culture is a more accessible method that can reveal the most dominant microorganism at an economic cost. In our large-scale study, we demonstrated a statistically significant difference between fungal and non-fungal rhinosinusitis. In the future, molecular and culture-based approaches should be combined to better understand the interactions between microorganisms and to develop microbiology-based treatment.

## 5. Conclusions

Fungal rhinosinusitis occurs more frequently in older patients and has a female predominance. Concurrent infections with *P. aeruginosa* are more commonly observed in patients with fungal rhinosinusitis. *P. aeruginosa* in sinonasal culture is an independent risk factor for recurrent disease after sinus surgery. By eliminating *P. aeruginosa*, we may prevent rhinosinusitis-related complications and reduce the risk of disease recurrence after sinus surgery in patients with fungal rhinosinusitis.

## Figures and Tables

**Table 1:** Demographic characteristics of fungal group and non-fungal group.

		Fungal Group		Non-Fungal Group	
Variables	*N*	(*N* = 898)	*N*	(*N* = 2884)	*p*-Value
Age (years)	898	56.9 ± 13.1	2884	47.0 ± 14.9	<0.001 **
Sex					<0.001 **
Male		338 (37.6%)		1817 (63.0%)	
Female		560 (62.4%)		1067 (37.0%)	
Sinusitis Lesion					<0.001 **
Unilateral		722 (80.4%)		1199 (41.6%)	
Bilateral		176 (19.6%)		1685 (58.4%)	
Nasal polyp		166 (18.5%)		1373 (47.6%)	<0.001 **
Allergy		57 (6.3%)		218 (7.6%)	0.423
Asthma		59 (6.6%)		189 (6.6%)	0.986
COPD		68 (7.6%)		214 (7.4%)	0.880
CKD		42 (4.7%)		63 (2.2%)	<0.001 **
ESRD		13 (1.4%)		24 (0.8%)	0.102
CVA		58 (6.5%)		120 (4.2%)	0.005 **
CAD		83 (9.2%)		125 (4.3%)	<0.001 **
Liver cirrhosis		12 (1.3%)		27 (0.9%)	0.300
Diabetes Mellitus		122 (13.6%)		215 (7.5%)	<0.001 **
Hypertension		218 (24.3%)		387 (13.4%)	<0.001 **

COPD = chronic obstructive pulmonary disease; CKD = chronic kidney disease; ESRD = end-stage renal disease; CVA = cerebrovascular accident; CAD = coronary artery disease. ** *p* < 0.01 (highly significant).

**Table 2:** Comparisons of lab data, need for revision surgery, and positive culture rate of *Pseudomonas aeruginosa* and *Staphylococcus aureus*.

	Fungal Group	Non-Fungal Group	
	(*N* = 898)	(*N* = 2884)	*p*-Value
Lab (Mean ± SD)			
WBC (1000/uL)	6.7 ± 1.9	7.5 ± 2.4	<0.001 **
Platelets (1000/uL)	246.3 ± 66.1	253.6 ± 68.0	0.005 **
Lymphocyte (%)	32.5 ± 8.9	30.8 ± 9.3	<0.001 **
Monocyte (%)	5.7 ± 1.9	5.6 ± 1.8	0.102
Basophil (%)	0.45 ± 0.28	0.47 ± 0.31	0.190
Segment (%)	59.1 ± 9.8	60.4 ± 10.3	0.002 **
Eosinophil (%)	2.2 ± 1.9	2.7 ± 2.4	<0.001 **
Revision surgery	32 (3.6%)	174 (6.0%)	0.004 **
Aerobic culture			
*P. aeruginosa*	128 (14.3%)	134 (4.6%)	<0.001 **
*S. aureus*	77 (8.6%)	351 (12.2%)	0.003 **
MRSA	16 (1.8%)	31 (1.1%)	0.095

WBC = white blood cell; *P. aeruginosa* = *Pseudomonas aeruginosa*; *S. aureus* = *Staphylococcus aureus*; MRSA = methicillin-resistant *Staphylococcus aureus*. ** *p* < 0.01 (highly significant).

**Table 3:** Odd ratios of fungal rhinosinusitis for multiple variables.

	Crude		Adjusted	
	OR (95% C.I.)	*p*-Value	OR (95% C.I.)	*p*-Value
Revision surgery				
No	Reference		Reference	
Yes	0.550 (0.359, 0.842)	0.006 **	0.670 (0.411, 1.092)	0.108
*P. aeruginosa*				
No	Reference		Reference	
Yes	3.392 (2.589, 4.444)	< 0.001 **	2.441 (1.781, 3.347)	<0.001 **
*S. aureus*				
No	Reference		Reference	
Yes	0.685 (0.520, 0.901)	0.007 **	0.726 (0.530, 0.994)	0.046 *

Odds ratio (OR) was adjusted for sex, age, comorbidities, and nasal polyps. *P. aeruginosa* = *Pseudomonas aeruginosa*; *S. aureus* = *Staphylococcus aureus*. * *p* < 0.05 (significant), ** *p* < 0.01 (highly significant).

**Table 4:** Characteristics of revision surgery subgroups in fungal group.

	Revision Surgery	Without Revision Surgery	
Variables	(N = 32)	(N = 866)	*p*-Value
Age (years)	48.7 ± 13.3	57.2 ± 13.0	<0.001 **
Sex			0.258
Male	9 (28.1%)	329 (38.0%)	
Female	23 (71.9%)	537 (62.0%)	
Sinusitis Lesion			0.216
Unilateral	23 (71.9%)	699 (80.7%)	
Bilateral	9 (28.1%)	167 (19.3%)	
Nasal polyp	8 (25.0%)	158 (18.2%)	0.334
Allergy	1 (3.1%)	56 (6.5%)	0.506
Asthma	1 (3.1%)	58 (6.7%)	0.986
COPD	2 (6.3%)	66 (7.6%)	0.880
CKD	1 (3.1%)	41 (4.7%)	<0.001 **
ESRD	0 (0.0%)	13 (1.5%)	0.102
CVA	2 (6.3%)	56 (6.5%)	0.005 **
CAD	2 (6.3%)	81 (9.4%)	<0.001 **
Liver cirrhosis	1 (3.1%)	11 (1.3%)	0.300
Diabetes Mellitus	2 (6.3%)	120 (13.9%)	<0.001 **
Hypertension	3 (9.4%)	215 (24.8%)	<0.001 **
WBC (1000/uL)	6.8 ± 1.9	6.7 ± 1.9	0.915
Platelets (1000/uL)	248.9 ± 64.2	246.2 ± 66.2	0.821
Lymphocyte (%)	34.9 ± 9.6	32.4 ± 8.9	0.156
Monocyte (%)	5.2 ± 1.5	5.8 ± 1.9	0.130
Basophil (%)	0.43 ± 0.30	0.45 ± 0.28	0.737
Segment (%)	56.4 ± 10.8	59.2 ± 9.8	0.158
Eosinophil (%)	3.1 ± 3.4	2.2 ± 1.8	0.196
Aerobic culture			
*P. aeruginosa*	9 (28.1%)	119 (13.7%)	<0.001 **
*S. aureus*	2 (6.3%)	75 (8.7%)	0.003 **

COPD = chronic obstructive pulmonary disease; CKD = chronic kidney disease; ESRD = end-stage renal disease; CVA = cerebrovascular accident; CAD = coronary artery disease; WBC = white blood cell; *P. aeruginosa* = *Pseudomonas aeruginosa*; *S. aureus* = *Staphylococcus aureus*. ** *p* < 0.01 (highly significant).

**Table 5:** Odd ratios of revision surgery in fungal group for multiple variables.

	Crude		Adjusted	
	OR (95% C.I.)	*p*-Value	OR (95% C.I.)	*p*-Value
Sex				
Male	0.599 (0.249, 1.442)	0.253	0.590 (0.215, 1.619)	0.305
Female	Reference		Reference	
Sinusitis lesion				
Unilateral	Reference		Reference	
Bilateral	1.245 (0.492, 3.152)	0.644	1.187 (0.420, 3.357)	0.747
Nasal polyp				
No	Reference		Reference	
Yes	1.375 (0.543, 3.487)	0.502	1.453 (0.514, 4.107)	0.482
*P. aeruginosa*				
No	Reference		Reference	
Yes	2.819 (1.196, 6.643)	0.018 **	4.056 (1.572, 10.460)	0.004 **
*S. aureus*				
No	Reference		Reference	
Yes	0.427 (0.057, 3.196)	0.407	0.436 (0.054, 3.546)	0.437

Odds ratio (OR) was adjusted for sex, age, comorbidities, and nasal polyps. *P. aeruginosa* = *Pseudomonas aeruginosa*; *S. aureus* = *Staphylococcus aureus*. ** *p* < 0.01 (highly significant).

## Data Availability

Restrictions apply to the availability of these data. Data were obtained from Chang Gung Research Database and are available with the permission of Institutional Review Board (IRB) of the Kaohsiung branches of Chang Gung Memorial Hospital.
